# A proteome map of primary cultured rat Schwann cells

**DOI:** 10.1186/1477-5956-10-20

**Published:** 2012-03-23

**Authors:** Mi Shen, Yuhua Ji, Shuqiang Zhang, Haiyan Shi, Gang Chen, Xiaosong Gu, Fei Ding

**Affiliations:** 1 Jiangsu Key Laboratory of Neuroregeneration, Nantong University, 19 Qixiu Road, Nantong, Jiangsu Province 226001, Peoples' Republic of China; 2 Institute of Tissue Transplantation and Immunology, College of Life Science and Technology, Jinan University, Guangzhou, China

**Keywords:** Schwann cell, Proteome, 2D LC/MS/MS

## Abstract

**Background:**

Schwann cells (SCs) are the principal glial cells of the peripheral nervous system with a wide range of biological functions. SCs play a key role in peripheral nerve regeneration and are involved in several hereditary peripheral neuropathies. The objective of this study was to gain new insight into the whole protein composition of SCs.

**Results:**

Two-dimensional liquid chromatography coupled with tandem mass spectrometry (2D LC-MS/MS) was performed to identify the protein expressions in primary cultured SCs of rats. We identified a total of 1,232 proteins, which were categorized into 20 functional classes. We also used quantitative real time RT-PCR and Western blot analysis to validate some of proteomics-identified proteins.

**Conclusion:**

We showed for the first time the proteome map of SCs. Our data could serve as a reference library to provide basic information for understanding SC biology.

## Background

Schwann cells (SCs) are the principal glial cells of the peripheral nervous system (PNS) with a wide range of biological functions [1]. Basically, SCs are able to wrap around the axons of neurons to form compact myelin sheaths, which allow for rapid and saltatory conduction of electrical impulses and support the integrity of axons in the PNS. SCs may also perform functions other than myelin formation; for example, SCs are involved in trophic support for neurons, formation of the neural extracellular matrix, modulation of neuromuscular synaptic activity, and orchestration of inflammation in the PNS. Collectively, SCs play a key role in the normal development and function of the PNS.

After peripheral nerve injury, SCs aid in cleaning up the tissue debris and guide the regrowth of axons. To accomplish this, SCs proliferate to form longitudinal cell strands known as bands of Bungner, release neurotrophins, and guide the regenerating axons to target organs. On the other hand, several hereditary peripheral neuropathies, such as Charcot-Marie-Tooth disease (CMT), Guillain-Barré syndrome (GBS), schwannomatosis, and chronic inflammatory demyelinating polyneuropathy (CIDP), are probably caused by genetic mutations in SCs, a knowledge of which is required for the prevention and treatment of these neuropathies.

Collectively, SC biology has been an active area of neuroscience research. As compared to genomic and transcriptomic analysis, the identification of the protein composition of SCs might be more valuable in examining the biological characteristics of SCs. Despite some subcellular studies on the proteomic profile of SC mitochondria in the disease state [2], there are few studies dealing with the comprehensive analysis of cellular proteins in SCs. In this study, therefore, we aimed to establish a proteome map of primary cultured SCs.

Proteomics strategy represents a powerful tool in the global investigation of a great multitude of cellular proteins, but the strategy has relied previously on two-dimensional gel electrophoresis (2-DE), which suffers from several drawbacks, including labor intensity, limited dynamic range, and an inability to detect hydrophobic, alkaline, and low abundance proteins under standard conditions [3,4]. The gel-based proteomics strategies are being replaced rapidly by a new proteomics strategy based on liquid chromatography coupled with tandem mass spectrometry (LC-MS/MS) [5-8], which shows a high degree of specificity and sensitivity.

By using 2D LC-MS/MS analysis, we identified a total of 1,232 proteins from primary cultured SCs and accomplished functional classification of the identified proteins. Obviously, the new insight into the protein composition of SCs not only contributes to the understanding of SC biology, but also provides an important basis for comparative studies between normal and diseased SCs.

## Results

### Isolation and characterization of primary cultured SCs

**Figure 1:**
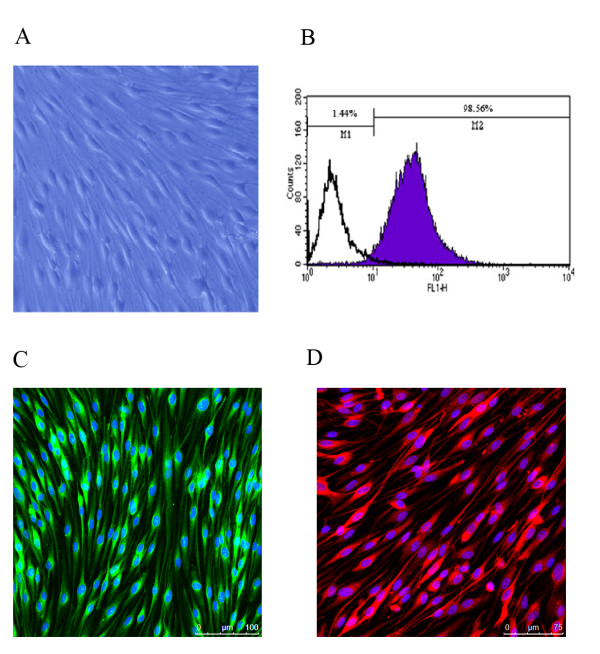
**Characterization of primary cultured SCs**. **(A) **Typical cell morphology of primary cultured SCs under phase-contrast microscopy (magnification 400×). **(B) **Representative flow cytometric analysis (FCA) data showing 98.56% of cell population was S100 β-positive. **(C) **S100 β (green color) immunocytochemistry combined with Hoechst 33342 staining (blue color) of primary cultured SCs. (D) GFAP (red color) immunocytochemistry combined with Hoechst 33342 staining (blue color) of primary cultured SCs.

For isolation and purification of SCs in vitro, we adopted efficient procedures to eliminate contamination of fibroblasts. The light micrograph demonstrated the typical morphology of primary cultured SCs (Figure 1A). The purity of primary cultured SCs was confirmed by flow cytometry data (Figure 1B), which indicated that 98.56% of the cell population was S100 β-positive (S100 β protein serving as a SCs marker). Immunocytochemistry with anti-S100 β and anti-GFAP provided further evidence for cell purity (Figure 1C and 1D).

### Identification, functional category, and subcelluar localization of cellular proteins in SCs

**Figure 2:**
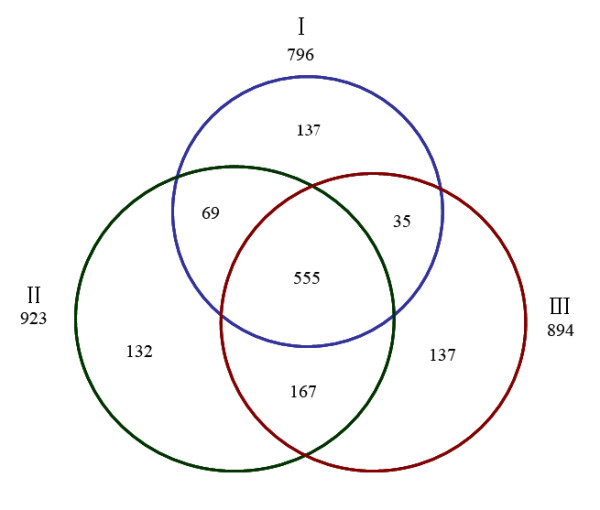
**The Venn diagram showing the overlap in protein identification among three independent proteomic analyses (I-III), which identified 796, 923, and 894 proteins, respectively**.

**Figure 3:**
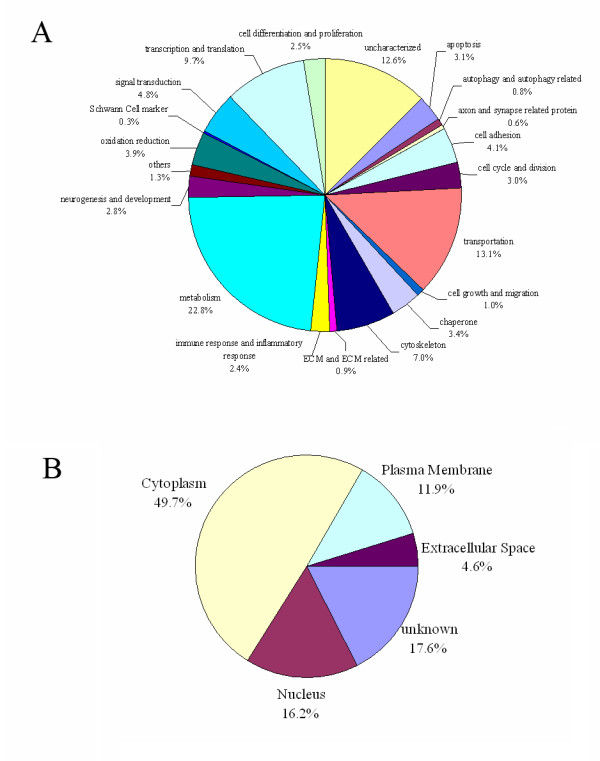
**Protein ontology**. Identified proteins were classified according to their biological functions **(A) **and subcellular localization **(B)**.

According to the criteria for protein identification, as mentioned in Materials and Methods, more than 700 proteins were identified in each independent biological replicate, and the corresponding false discovery rates (FDRs) of three individual analyses were less than 1%. Detailed information on identified peptides and proteins is provided in Additional file 1. Subsequently, proteins identified from three independent analyses and proteins under identical accession number and/or gene symbol were merged. The total number of proteins identified in this study was 1,232. Out of 1,232 proteins, 846 were identified by two or more unique peptides and the remaining 386 were identified by one unique peptide. The annotated spectra of proteins identified on the basis of one unique peptide are provided in Additional file 1. The Venn diagram (Figure 2) shows that among 1,232 identified proteins, 555 (45%) were shared by all three experiments, and 271 (22%) were shared by two experiments (Figure 2); thus, 67% of the proteins were identified by more than one experiment, confirming the good reproducibility of the adopted proteomics platform in protein identification. Furthermore, the 1,232 identified proteins were categorized into 20 different classes in terms of their main biological functions by searching the UniProt protein knowledge database http://www.uniprot.org/ and PubMed http://www.ncbi.nlm.nih.gov/pubmed/ (Figure 3A), and the details are provided in Additional file 1. The identified proteins were further assigned to various subcellular compartments of SCs using ingenuity pathway analysis (IPA) software (Figure 3B). The results of subcelluar localization of the 1,232 proteins are listed in Additional file 1. It was noted that almost half of the proteins were located in the cell cytoplasm.

### Validation of protein expressions

**Figure 4:**
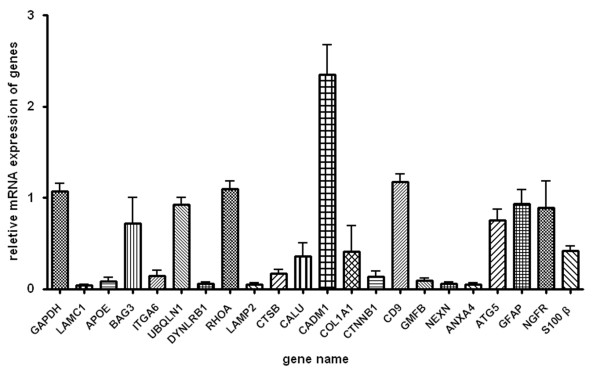
**Validation by qPCR**. Histogram showing the mRNA expression level of LAMC1, APOE, BAG3, ITGA6, UBQLN1, DYNLRB1, RHOA, LAMP2, CTSB, CALU, CADM1, COL1A1, CTNNB1, CD9, GMFB, NEXN, ANXA4, ATG5, GFAP, NGFR and S100 β, relative to that of GAPDH, in primary cultured SCs, as determined by qPCR.

Out of the total 1,232 proteins, 21 proteins, including LAMC1, APOE, BAG3, ITGA6, UBQLN1, DYNLRB1, RHOA, LAMP2, CTSB, CALU, CADM1, COL1A1, CTNNB1, CD9, GMFB, NEXN, ANXA4, ATG5, GFAP, NGFR and S100 β, were subjected to further validation by quantitative real time RT-PCR (qPCR), and the mRNA expression levels of these proteins are shown in Figure 4.

**Figure 5:**
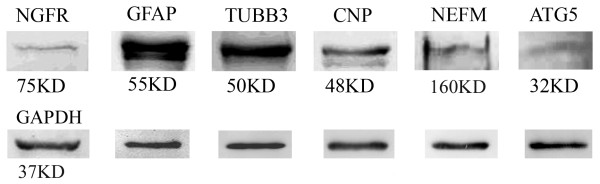
**Validation by Western blot analysis**. The representative Western blot image showing the protein expression of NGFR, GFAP, TUBB3, CNP, ATG5 and NEFM in primary cultured SCs. Equivalent amounts of protein were loaded per gel. GAPDH was used as the protein loading control for each blot.

**Figure 6:**
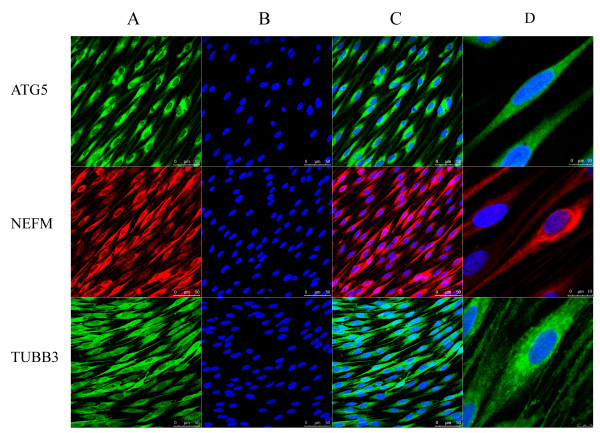
**Validation by immunocytochmistry**. Light micrographs, taken by a TCS SP5 confocal microscope equipped with a 63 × oil immersion lense, showed that **(A) **primary culture of SCs were immunostained by antibodies against ATG5 (green color), NEFM (red color), or TUBB3 (green color), respectively; **(B) **the cell nuclei were stained by Hoechst 33342 (blue color); **(C) **the merge of (A) and (B); and **(D) **the views at a much higher magnification of one cell each per antigen. Scale bar as indicated in the images.

The other 6 identified proteins, CNP, GFAP, NGFR, TUBB3, ATG5 and NEFM, were validated by Western blot analysis (Figure 5). Among these 6 proteins, 3 proteins, TUBB3, ATG5 and NEFM, were further subjected to immunocytochemistry (Figure 6).

In all the validated proteins, GFAP, NGFR and S100 β are markers of SCs [9]; LAMC1, APOE, CTNNB1, CD9, CNP, and ITGA6 have been reported to be expressed in SCs [10-16]; but the existence of other proteins in SCs has been little reported to our knowledge.

## Materials and methods

### Cell culture

The rat SCs were harvested as previously described [17] with minor modifications. Briefly, sciatic nerves were harvested from Sprague-Dawley rats (1 to 3 d- old) and enzymatically dissociated by incubation at 37°C sequentially with 1% collagenase and 0.125% trypsin for 30 and 10 min, respectively. The mixture was triturated, centrifuged and resuspended in 10% FBS in DMEM. The cell pellets were plated on poly-L-lysine precoated dishes (35 mm) for incubation in the same medium. On the following day, 10 μM cytosine arabinoside was added and allowed to incubate for an additional 48 h to remove fibroblasts. The cell culture was maintained subsequently in DMEM supplemented with 10% FBS, 2 μM forskolin (Sigma, St Louis, MO) and 2 ng/ml heregulin (HRG, Sigma) to stimulate SC proliferation. For further purification, the cell culture was gently trypsinized, pelleted, and incubated with anti-Thy1 antibody (AbD Serotec, Raleigh, NC) on ice for 2 h, followed by incubation in complement (Jackson Immuno, West Grove, PA) for an additional 2 h. All media and supplements were bought from Gibco-Invitrogen (Carlsbad, CA).

### Immunocytochemistry

After isolation and purification, primary cultured SCs were subjected to immunocytochemistry with anti-S100β, anti-GFAP, anti-TUBB3, anti-NEFM or anti-ATG5. Briefly, the cell culture was fixed in 4% paraformaldehyde (pH 7.4) for 30 min, permeabilized with 0.3% Triton X-100, 10% goat serum in 0.01 M phosphate buffered saline (pH 7.2) for 60 min at 37°C, and allowed to incubate with anti-S100β, anti-GFAP (1:200, Abcam, Cambridge, MA), anti-ATG5 (1:200, Abgent INC, San Diego, CA) and anti-TUBB3, anti-NEFM (1:200, Sigma) antibody respectively at 4°C overnight, followed by reaction with FITC- or PE-conjugated goat anti-rabbit IgG (1:400, Molecular probes, the Netherlands) for 2 h at room temperature, respectively. The cells were also stained with 5 μg/ml Hoechst 33342 dye at 37°C for 10 min. The fluorescence was visualized under a TCS SP5 confocal microscope (Leica Microsystems, Wetzlar, Germany).

### Flow cytometry

For flow cytometric analysis (FCA), primary cultured SCs were dissociated by treatment with 0.125% (w/v) trypsin, and the cell pellets were resuspended in a fixation medium (Invitrogen, Carlsbad, CA) and incubated for 15 min at room temperature. Permeabilization Medium (Invitrogen, Carlsbad, CA) and the recommended volume of the anti-S100β antibody were added (Abcam, Cambridge, MA) to allow incubation for 20 min. Cells were then stained with CFTM488A IgG secondary antibodies (Biotium, Hayward, California) at room temperature for 30 min. Flow cytometric acquisition and data analysis were performed with a flow cytometer and cellquest software (BD FACScalibur, BD Bioscience, San Jose, CA). As a negative control, the cells were incubated only with the FITC-conjugated secondary antibody. Three independent flow cytometric experiments were performed.

### Sample preparation

Cell cultures were washed with ice-cold phosphate buffered saline (PBS) and lysed in a buffer containing 50 mM Tris-HCl (pH7.6), 5 mM EDTA, 50 mM NaCl, 30 mM sodium pyrophosphate, 50 mM NaF, 0.1 mM Na_3_VO_4_, 1% (v/v) Triton X-100, 1 mM PMSF, and a protease inhibitor mixture tablet (Roche Applied Science, Mannheim, Germany). Lysates were clarified by centrifugation at 15,000 × g for 20 min at 4°C, and protein concentration was determined by Bradford protein assay (Bio-Rad, Richmond, CA).

### Digestion, sample cleaning, and desalting

Protein from primary cultured SCs was precipitated with ice-cold acetone overnight at -20°C, and pellets were dissolved, denatured, alkylated and digested with trypsin (1:20, w/w, Sigma) at 37°C for 18 h. Prior to on-line 2D nano LC/MS/MS analysis, samples were cleaned and desalted. A cation exchange cartridge system (Applied Biosystems, Foster city, CA) was used to remove the reducing reagent, SDS, undigested proteins, and trypsin in the sample mixture because these materials would interfere with the LC/MS/MS analysis. Subsequently, the eluate of cation exchange was desalted on a 4.6-mm-inner diameter × 150-mm C_18 _reversed-phase column (5 μm, 80 Å; Agilent, Waldbronn, Germany).

### On-line 2D nano LC/MS/MS

2D nano LC/MS/MS analyses were conducted on a nano-HPLC system (Agilent, Waldbronn, Germany) coupled to a hybrid Q-TOF mass spectrometer (QSTAR XL, Applied Biosystems) equipped with a nano-ESI source (Applied Biosystems) and a nano-ESI needle (Picotip, FS360-50-20; New Objective Inc., Woburn, MA). Analyst™ 1.1 software was used to control the QSTAR XL mass spectrometry and nano-HPLC system and to acquire mass spectra. Vacuum-dried peptides were reconstituted in phase A and injected at a flow rate of 10 μl/min onto a high resolution strong cation exchange (SCX) column (Bio-SCX, 300-μm inner diameter × 35 mm; Agilent, Wilmington, DE), which was on line with a C_18 _precolumn (PepMap, 300-μm inner diameter × 5 mm; LC Packings). After loading, the SCX column and C_18 _precolumn were flushed with a 16-step gradient sodium chloride solution (0, 10, 20, 30, 40, 50, 60, 70, 80, 90, 100, 125, 150, 200, 300, and 400 mM) for 5 min and phase A for 10 min at a flow rate of 15 μl/min. Afterwards, the precolumn was switched on line with a nanoflow reversed-phase column (VYDAC 218MS, 75-μm inner diameter × 100 mm; Grace, Hesperia, CA), and the peptides concentrated and desalted on the precolumn were separated using a 120-min linear gradient from 12 to 30% (v/v) phase B (0.1% (v/v) FA in acetonitrile) at a flow rate of 400 nl/min.

The Q-TOF instrument was operated in a positive ion mode with ion spray voltage typically maintained at 2.0 kV. A mass spectrum of the sample was acquired in an information-dependent acquisition mode. The analytical cycle consisted of a 0.7-s MS survey scan (400-1600 m/z) followed by three 2-s MS/MS scans (100-2000 m/z) of the three most abundant peaks (*i.e*. precursor ions), which were selected from the MS survey scan. Precursor ion selection was based upon ion intensity (peptide signal intensity above 25 counts/s) and charge state (2+ to 4+), and once the ions were fragmented in the MS/MS scan, they were allowed one repetition before a dynamic exclusion for a period of 120 s. Under collision-induced dissociation (CID), fragment ions of the peptides were produced, resulting in sequencing of the peptides and identification of the corresponding proteins. External calibration of mass spectrometer was carried out routinely using reserpine and trypsinized bovine serum albumin.

### Protein identification

The complete set of raw data files (*.wiff) of each run were uploaded to ProteinPilot software 3.0 (Applied Biosystems) and searched against the non-redundant International Protein Index (IPI) rat sequence database (version 3.62, 39,867 entries). The search parameters were as follows: trypsin digestion; methyl methane thiosulfate alkylation of cysteine residue, instrument, QSTAR ESI; identification focus, biological modifications, and FDR analysis selected. ProteinPilot uses Paragon algorithm for peptide identification and ProGroup algorithm to assemble the peptide evidence from the Paragon algorithm to find the smallest number of proteins that could explain all the fragmentation spectral evidence. Protein identification is based on the Unused ProtScore score, which is a measurement of all the peptide evidence for a protein that is not better used by a higher-ranking protein. In this study, the identification of a protein was reported for unique peptides with an "unused" confidence threshold (ProtScore) of > 1.3%, and with a corresponding FDR of less than 1%.

### Functional category and localization of identified proteins

To obtain an overview of their biological significance, the identified proteins were categorized according to their main biological functions collected from the Uniprot protein knowledge database and PubMed. The localization of the proteins was analyzed by Ingenuity pathway analysis (IPA, http://www.ingenuity.com).

### qPCR

Total RNA was extracted from the primary cultured SCs using Trizol (Invitrogen), and cDNA was synthesized from the total RNA using the SuperScript First-Strand Synthesis System (Invitrogen). The qPCR was conducted by FastStart^® ^SYBR Green qPCR Master Mix (Roche, Germany) according to the manufacture's specifications. A 50-μl reaction consisted of 1 μl of cDNA, 25 μl of 2 × Fast SYBR Green Master Mix, 1 μl of each primer, and 22 μl of RNase/DNase free water. Two-step fast cycling protocol was used in StepOne™ Real-Time PCR System (Applied Biosystems), and the data were analyzed using the software supplied by the vendor (Applied Biosystems). Primer sequences are reported in Additional file 2.

### Western blot analysis

Cell proteins were extracted from primary cultured SCs and quantified by a BCA kit. Samples containing 15 μg of total protein were separated by 12% (w/v) SDS-PAGE and transferred to a PVDF membrane (Millipore, Bedford, MA). After incubation for 1 h in 5% (w/v) nonfat milk in TBS-T buffer, the membrane was probed with the indicated primary antibodies overnight at 4°C. After wash with TBS-T, the membrane was incubated with IRDye 800-Conjugated secondary antibodies (Odyssey) for 1 h at room temperature. The images were scanned with the GS800 densitometer scanner (Bio-Rad, Hercules, CA). The primary antibodies used were rabbit polyclonal antibody against CNP or GFAP (Bioworld Technology Inc. Louis Park, MN), rabbit polyclonal antibody against NGFR (Abcam UK), goat polyclonal antibody against TUBB 3, mouse monoclonal antibody against NEFM (Sigma), and rabbit polyclonal antibody against ATG5 (Abgent, San Diego, CA).

### Statistical analysis

Data are presented as the mean ± S.D. Data comparison was performed by unpaired Student's *t*-test with SPSS10.0 software. Statistical significance was set at p < 0.05. Unless otherwise specified, all assays were conducted at least in triplicate.

## Discussion

There have been several proteomic studies concerning the central nervous system (CNS), providing us with considerable information in the field of neuroproteomics [18-28]. In contrast, SCs, as one of the key components in the PNS, have received little research attention from the perspective of proteomics. A pioneer study accomplished proteome analysis of PNS myelin, which, formed by SCs, is comprised of multiple compacted layers of the molecularly specialized plasma membrane extended from SCs [29]. This study focused on the large-scale proteomics analysis of SCs themselves, which has hardly been reported before.

Metabolism-related proteins represented the largest class (22.8%) of proteins expressed in SCs. For example, APOE (apolipoproteinE), a lipid metabolism-related protein, has been shown to protect mice from chronic inflammatory demyelinating polyneuropathy by affecting the antigen-presenting function of SCs [11], and to benefit axonal reconstruction and myelin membranes [30]. It follows that this type of protein is responsible for some of the biological characteristics of SCs.

Other identified proteins are respectively related to a wide range of functions, including transportation (13.1%), transcription and translation (9.7%), cytoskeleton (7.0%), signal transduction (4.8%), cell adhesion (4.1%), oxidation reduction (3.9%), chaperone (3.4%), apoptosis (3.1%), cell cycle and division (3.0%), neurogenesis and development (2.8%), cell differentiation and proliferation (2.5%), immune and inflammatory response (2.4%), cell growth and migration (1.0%), ECM and ECM related (0.9%), autophagy and autophagy related (0.8%), axon and synapse related protein (0.6%), and cell markers (0.3%). In addition, we also found several proteins with miscellaneous (1.3%) or uncharacterized (12.6%) functions.

Intriguingly, autophagy and autophagy-related proteins were expressed in SCs. Autophagy, as a catabolic process for the autophagosomic-lysosomal degradation of bulk cytoplasmic contents, is generally activated by conditions of nutrient deprivation, and is also associated with a diverse array of physiological and pathological processes, including development, differentiation, infection, cancer and neurodegeneration [31]. The identification of conserved autophagy genes (ATGs) suggests the existence of autophagy [32], and autophagy upregulation may assist in curing diseases caused by toxic intracellular aggregate-prone proteins or may serve as a lifespan extender in the normal body [33]. Previous studies have shown that glial cells from neuropathic mice activate autophagy in response to rapamycin and produce abundant myelin internodes, and that both the ubiquitin-proteasome system and autophagy mediate the trauma-induced axonal degeneration and the retrieval of nerve growth factors [34,35]. In this study, we detected a class of autophagy-related proteins, including ATG3, ATG5, LAMP1, and UBQLN1, in primary cultured SCs. As is known, ATG5 protein contributes to inhibiting lethal Sindbis virus infections of the CNS in mice [36] and is involved in immune-mediated myelin injury in mice [37]; disruption of autophagy by mutation of ATG5 or ATG7 causes neurodegeneration [38]. In our study, qPCR, Western blot and immunohistochemistry confirmed the expression of the ATG5 protein in primary cultured SCs. Therefore, we assumed that the expressions of ATG5 and other autophagy-related proteins in SCs might influence the PNS. Another interesting finding of our confirmatory tests is that some proteins generally considered to be expressed in neurons, such as TUBB3 (tubulin3 β chain), TUBB2 (tubulin2 β chain), and NEFM (neurofilament medium polypeptide), were also present in SCs. Previous studies have reported the mRNA expression of NEFM and tubulin in SCs and TUBB3 expression in neurofibroma SCs [39,40]. To a certain degree, our results provided further evidence for these previous studies.

## Conclusion

In this study, the 2D LC/MS/MS approach illustrated the proteome map of primary cultured SCs. We identified a total of 1,232 proteins, among which, 846 were identified by two or more unique peptides while the remaining 386 were detected by one unique peptide. Our data could be used as a reference library to provide basic information for studying SC biology.

## Competing interests

The authors declare that they have no competing interests.

## Authors' contributions

MS carried out experiments and data analysis, and composed the draft. YJ carried out the proteomic analysis, proteomic data acquisition and interpretation. SZ performed Western blot analysis. SZ, HS and GC contributed to proteomic data analysis. XG contributed to the project idea and obtained grant funding. FD contributed to the concept and design of the project, manuscript preparation, and obtained grant funding. All authors have read and approved the final manuscript.

## Supplementary Material

Additional file 1 **The protein list of three independent experiments, biological function categories and subcellular localization of 1,232 proteins, and annotated spectra of proteins identified on the basis of one unique peptide spectrum**. Unused (ProtScore): a measurement of the protein confidence for a detected protein, calculated from the peptide confidence for peptides from spectra that have not already been completely "used" by higher scoring winning proteins. Total (ProtScore): a measure of the total amount of evidence for a detected protein. % Cov (95): The percentage of matching amino acids from identified peptides having confidence greater than or equal to 95 divided by the total number of amino acids in the sequence. (From Proteinpilot online help). Click here for file

Additional file 2 **The 22 primer sequences used in qPCR**. Click here for file
